# *“Life at the River is a Living Hell:*” a qualitative study of trauma, mental health, substance use and HIV risk behavior among female fish traders from the Kafue Flatlands in Zambia

**DOI:** 10.1186/s12905-017-0369-z

**Published:** 2017-03-07

**Authors:** Lynn T. Murphy Michalopoulos, Stefani N. Baca-Atlas, Simona J. Simona, Tina Jiwatram-Negrón, Alexander Ncube, Melanie B. Chery

**Affiliations:** 1 Columbia University School of Social Work, Social Intervention Group, Global Health and Mental Health Unit, 1255 Amsterdam Avenue, Room 804, Mail Code 4600, New York, NY 10027 USA; 2 University of North Carolina at Chapel Hill School of Social Work, Chapel Hill, North Carolina USA; 3 Department of Social Development Studies, University of Zambia, School of Humanities and Social Sciences, Lusaka, Zambia; 4 University of Michigan, Curtis Center, School of Social Work, 1080 South University, Ann Arbor, 48109-1106 Michigan USA; 5 Elizabeth Glaser Pediatric AIDS Foundation, Zambia, Lusaka, Zambia; 6 Columbia University School of Social Work, New York, NY 10027 USA

**Keywords:** Female fish traders, Trauma, Mental health, HIV sexual risk, Zambia, Transactional sex, Qualitative methods

## Abstract

**Background:**

In Western settings, the relationship between trauma history, posttraumatic stress disorder, substance use, and HIV risk behavior, is well established. Although female fish traders in Zambia are affected by HIV at rates estimated to be 4–14 times higher than the national prevalence, no studies have examined the co-occurring issues of trauma, substance use and HIV risk behavior among this vulnerable population. The current study examined: 1) trauma history, trauma symptoms and HIV risk behaviors and 2) the relationship between these co-occurring issues among female fish traders from the Kafue Flatlands in Zambia.

**Methods:**

Twenty individual semi-structured qualitative interviews and a focus group discussion (*n* = 12 participants) were conducted with female fish traders in the Kafue Flatlands of Zambia. Template analysis was used to examine the data.

**Results:**

The findings indicate that female fish traders in Zambia are at risk of multiple and ongoing traumatic events and daily stressors, severe mental health symptoms (including western conceptualizations of disorders such as anxiety, depression, post-traumatic stress disorder (PTSD) and complicated grief, as well as local idioms of distress), substance abuse, and HIV sexual risk behaviors. The results suggest a relationship between trauma and HIV sexual risk behavior in this population.

**Conclusions:**

The indication of these co-occurring issues demonstrates the need for HIV prevention intervention efforts, which account for trauma, mobility, and psychosocial outcomes in order to reduce HIV sexual risk behavior among female fish traders in Zambia.

**Electronic supplementary material:**

The online version of this article (doi:10.1186/s12905-017-0369-z) contains supplementary material, which is available to authorized users.

## Background

In sub-Saharan African, “fisher folk” (i.e., individuals who directly or indirectly derive their livelihood from the fishing industry) have been noted as a key-affected population for Human Immunodeficiency Virus (HIV), with prevalence rates 4–14 times higher than national rates [1–4]. “Fisher folk” are potentially at high risk for HIV due to lack of accessible health services and lengthy time away from home [2, 5, 6]. Female fish traders, who buy fish from fishermen, process the fish and then travel long distances to sell the product in markets, are particularly at risk for HIV infection [4, 7]. Transactional sex during the process of transportation to- and from fishing camps with truck drivers, “fish for sex” negotiations with fishermen, and the subordinate social and economic position of women in fishing communities in sub-Saharan Africa are primary drivers of HIV risk among female fish traders [1, 4, 8, 9]. Similar to occupations of other labor migrants from low and middle income countries (LMIC), the female fish trading business has been labeled a 3-D job (i.e., dangerous, demeaning and difficult) [8]. Yet, no studies in sub-Saharan Africa, to date, have examined the psychosocial outcomes related to trauma exposure or the ongoing daily stressors related to this highly gendered, stressful, and dangerous occupation among women. Further, no studies have examined the relationship between co-occurring work-related traumatic events, psychosocial outcomes, and HIV risk among this key-affected population.

### HIV risk among female fish traders in Zambia

Zambia is heavily burdened with HIV, with 13% of the population, aged 15–49 being infected [10]. While HIV studies among female fish traders in Zambia are limited, evidence suggests even higher rates of HIV infection among this population. One study found an HIV prevalence of 24% or higher in the fishing community of the Kafue flatlands [11]. Meanwhile, fish accounts for over 20% of animal protein consumed, providing essential nutrients for a country with high rates of malnutrition [12]. Further, in 2006, it was estimated that over 1 million people, or 7% of the population, in Zambia rely on the fishing industry for income [5, 11, 13]. Given the high reliance on fish and the fishing industry as well as the burden of HIV among fisher folk, understanding the drivers of HIV risk behavior is critical to addressing health concerns, and essential for productive and sustainable fisheries in Zambia [5, 8].

The fishing industry in sub-Saharan Africa involves highly structured gender-roles. Women most commonly engage in the trade and sale of fish [4, 7, 14], receiving lower pay than their male counterparts, who are primarily involved in catching fish. The resulting structure is a reliance of female fisher folk on male fisher folk for resources. Combined with a lower socio-economic positioning, Zambian female fish traders with AIDS-related illnesses suffer from additional challenges: they are less able to meet the physical demands of fish trading, such as drying the fish or transporting fish with ice blocks across long distances [5]. In addition, with limited access to health services [4, 5], female fish traders living with HIV are at even greater risk for poor health outcomes and mother-to child transmission of HIV. Further, engaging in transactional sex with truck drivers and fishermen [1, 2, 8] has the potential to spread HIV not only throughout Zambia, but through surrounding countries in southern Africa given migration patterns of fisher folk.

### Current state of the literature and gaps

Research examining factors associated with HIV risk behavior among female fish traders in sub-Saharan Africa is scarce, but emerging research indicates growing awareness of the public health implications of HIV among this population. Research suggests several drivers of risk including substance use, gendered dynamics, social and economic vulnerabilities, and violence. For example, a recent longitudinal study by Seeley and colleagues [15] found that alcohol and marijuana use, as well as a history of a sexually transmitted infection was associated with HIV infection rates among fisher folk in Uganda. Also in Uganda, Sileo, Kintu, Chanes-Mora and Kiene [16] found that heavy alcohol use associated with sexual risk behavior was common among fisher folk. Further, MacPherson and colleagues [4] found that transactional sex, economic need and structured gender-based relationships decreased the ability of female fish traders in Malawi to negotiate condom use. Additional research suggests that female fish traders have been highly stigmatized by their communities and held responsible for the spread of AIDS in fishing communities and surrounding areas in sub-Saharan Africa [8, 9]. In a fishing community of Tanzania, transactional sex, mobility and a lack of access to condoms was found to be related to HIV infection [17]. In Zambia, Husken and Heck [18] found that female fish traders are at risk of violence due to the necessary reliance on interactions and relationships with fishermen, with over 15% indicating forced sex for fish interactions, increasing HIV risk [18].

Although previous literature has examined sexual violence and substance use among female fish traders in sub-Saharan Africa, there are no known studies in the extant literature that have examined exposure to migrant-related trauma, ongoing daily stressors, and subsequent psychosocial outcomes among female fish traders in Zambia. Further, no known studies have examined culturally-specific trauma outcomes which are not limited to western conceptualizations of mental health symptoms (e.g. post-traumatic stress disorder (PTSD), depression) among this population. Understanding trauma exposure, outcomes of trauma and the potential relationship to HIV risk behavior is critical in order to develop relevant and culturally appropriate HIV prevention interventions among female fish traders. As the relationship between trauma history, PTSD, substance use, and HIV sexual risk behavior has been well established among different key populations in Western settings [19–21], this must be explored among female fish traders in Zambia, a vulnerable population in an environment where there is potential for multiple and ongoing traumatic events and known high HIV prevalence.

### Conceptual model

The current study was guided by the sensitizing concepts that comprise the theoretical framework put forth by Wingood and DiClemente [22], the *theory of power and gender on women’s health*. An adaptation of Connell’s work [23], the theory holds that there are three structures, which contribute to HIV risk among women: the sexual division of labor, the sexual division of power, and the structure of cathexis related to gender norms and roles [22]. Applied to female fish traders in Zambia, socioeconomic, behavioral, and personal factors may influence risk of HIV acquisition and transmission. Specifically social exposures, such as lower social status as well as witnessed or experienced violence and personal risk factors, such as substance use and mental health are of central focus. Couched within this framework, the current study explored the sensitizing concepts of personal risk factors from the conceptual model by Miller [24], which explains the pathway from sexual violence to HIV sexual risk behavior among women at the individual level. Grounded in our participants’ experiences, we explored exposure to migrant-related trauma and ongoing daily stressors as increasing the risk of mental health symptoms and substance use contributing to HIV sexual risk behavior among female fish traders. Trauma survivors often experience ongoing feelings of shame, guilt, and fear, lack self-efficacy, and are at risk of developing symptoms of PTSD [25]. An increase in trauma symptoms and a decrease in one’s sense of control can increase the use of maladaptive coping mechanisms such as substance use and risky sexual behavior, potentially leading to the spread of HIV [24, 26, 27]. Substance use can also increase the severity of symptoms of depression and post-traumatic stress disorder [24, 28] and can place women at risk for sexual violence [29]. As such, the purpose of this paper is to explore 1) the extent and types of trauma exposure among female fish traders in Zambia 2) outcomes, including both western conceptualizations of mental health and substance use, as well as “local” trauma symptoms, experienced as a result of psychosocial stressors and trauma 3) the relationship between trauma/migrant-related stressors, mental health problems, substance use and HIV sexual risk behavior among female fish traders in Zambia.

## Methods

### Study site

This study was located in the Kafue Flatlands in Kafue district and John Leinge Fish Depot in Lusaka. The Kafue flatlands are a flood plain of 6,500 km^2^ both in the south and north bank of the Kafue river, located approximately 200 km southwest of the capital, Lusaka. The area is inhabited mainly by the Ila and plateau Tonga agro-pastoralists, but the study focused on women migratory fish traders sheltered in seasonal camps in the woodlands bordering the floodplains and in the floodplains during the dry season. The site was selected for the study not only because of the existence of large-scale fishing activities, but also its relative proximity to Lusaka, a main re-selling point. John Leinge Fish Depot is located around 4 km south of Lusaka’s central business district. The Depot was selected as it is the largest selling point for Kafue fish traders.

### Study design

This study was conducted between August 2014 and January 2015. The study design was qualitative and included individual semi-structured interviews and a focus group discussion (FGD) to confirm and complement the findings from the interviews. The primary role of the FGD was for member checking purposes (i.e., testing our interpretation of the data for validity specifically among female fish traders) [30]. However, when participants during the FGD shared additional information relevant to the research questions, those data were incorporated into the analyses.

A qualitative approach was used for a number of reasons. First, there are no studies, to date, that examine the local understanding and relevance of trauma outcomes, including western conceptualizations of mental health (e.g. post-traumatic stress disorder, depression, substance use), which may be applicable cross-culturally, and/or specific to Zambian female fish traders. Second, empirical research on the relationship between trauma, trauma outcomes, and HIV risk behavior has been limited in LMIC among migrant populations [31], and therefore should be explored as a relevant relationship in LMIC, through methods that allow for the unique perspective and individual experiences to be expressed. As an extremely disenfranchised, understudied, and vulnerable population, qualitative methodology was critical to obtaining the lived experience of female fish traders in Zambia. Finally, qualitative methods are an appropriate first step to inform future quantitative methods.

The study relied heavily on community engagement and local partners to: 1) establish trust with the female fish traders, 2) ensure cultural relevance and 3) begin the formation of relationships for the sustainability of future potential HIV intervention preventions. A local male research assistant conducted the semi-structured interviews in Nyanja or Bemba, local Zambian languages. Given the sensitive nature of the questions posed to participants, a female interviewer would have been ideal. However, a male interviewer was chosen because of his background in qualitative interviewing and familiarity with the context and language as a local Zambian. The focus group discussion was conducted by a local fish trader community mobilizer and local partner for the study from the Ministry of Community Development and Maternal and Child Health. The FGD was conducted in Nyanja.

The semi-structured interview guides covered key content areas including: Living Arrangements; Migration and Mobility Experience; Employment and Socializing; Views on Relationships, Sex, and Intimacy; HIV/AIDS/STI Awareness; HIV risk behaviors; Mobility and Sexual Experiences; Migrant-Related Stressful Life Events/Trauma; Psychosocial Problems related to trauma/stressors; Ways of Coping with Problems. The FGD interview guide was developed using preliminary findings from the semi-structured interviews.

In order to assess non-western or “local” conceptualizations of psychosocial/mental health outcomes experienced by female fish traders, participants were asked to describe any problems including thoughts, feelings or behaviors experienced as a result of experiencing a traumatic/stressful event, witnessing another female fish trader’s experience, or hearing of a female fish trader experience. After probing for local conceptualizations of mental health, fish traders were also asked if they experienced any of the symptoms of post-traumatic stress disorder, according to the 5^th^ edition of the Diagnostic and Statistical Manual [32]. For each endorsed symptom of PTSD symptom, fish traders were asked to provide a description or example of the specific item.

Interview guides were translated to Nyanja and Bemba by a certified translator at the University of Zambia and back-translated to English by another certified translator at the University of Zambia. The guides were then pre-screened and reviewed for feedback and relevance with the study team including the local research coordinator, officials from the Ministry of Community Development and Maternal and Child Health, and fish trader community mobilizers.

### Data collection

Data collection for the individual semi-structured interviews occurred between August and October 2014. The FGD was held in January 2015. A purposive sampling strategy was utilized for the current study. For the semi-structured interviews, female fish traders at the fishing camp in Kafue Flatlands were approached by the research coordinator and a female fish trader community mobilizer. For the FGD, female fish traders at the market of the John Lainge Fish Depot were approached by the research coordinator and a community mobilizer. Inclusion criteria included (all by self-report): being 17–65 years old; employed as a female fish trader; being a citizen of Zambia AND maintaining a permanent residence that is beyond commuting distance to the study site OR travels beyond commuting distance from the study site for fish trading work; and able to speak Nyanja or Bemba fluently. Twenty semi-structured interviews and one FGD (including 12 female fish traders not previously interviewed) were conducted for the current study. Previous qualitative research has suggested that saturation occurs after 10–15 interviews [33]. As this was cross-cultural research with an understudied population, an additional 5 interviews (*N* = 20 total) were conducted to ensure saturation.

### Data analysis

Data analysis for the current study was informed by the template analysis approach [34, 35]. Template analysis is a structured technique for analyzing qualitative data, allowing the researcher to establish order of the data from the beginning of the analytic process, based on the research question and a priori codes [34]. Hierarchical coding is emphasized within template analysis, using broad themes that successively become narrower. This approach is also flexible in that any of the main or sub-themes identified can be modified or removed if they do not prove to be useful for the data [34]. Finally, the approach allows for emergent themes in response to the data, which may not have been identified from the original ‘template’ [35].

To increase trustworthiness and enhance cultural accuracy of data collected, a number of steps were taken. First, three members of the research team (LM, SB, and MC) separately conducted an initial review of the data from the semi-structured interviews. As a group, the research team then discussed the relevance of each *a priori* code and any emergent codes. Where there was a discrepancy, the group discussed the data until consensus was reached. Second, all sub-themes from the interviews and FGD that were identified, were reviewed by a local research partner in Zambia (SS) to ensure cultural relevance and accuracy. Finally, a FGD was conducted as member checking to obtain respondent feedback and clarify any themes/sub-themes from the preliminary findings.

## Results

### Demographics

Demographic information of participants from the semi-structured interviews and FGD are listed in Table I. The majority of participants were married (*N* = 14), or widowed (*N* = 10). The average education completed among all participants was grade 7. Participants from the interviews reported working as a female fish trader for many years, with an average of over 16 years. Most of the participants reported a negative HIV status, while 20% reported an unknown HIV status (see Table 1). The raw demographic data can be accessed in the Additional file 1.

**Table 1:** Demographics of Semi-Structured Interviews and Focus Group Discussion

Demographic Variable	Semi-Structured Interviews	Focus Group Discussion
Total Sample	*N* = 20	*N* = 12
Age	M = 40.7 years	M = 32.2 years
	(range = 23-63 years.)	(range = 17-55 years.)
	(IQR = 8.5)	(IQR = 17)
Marital Status		
Married	*N* = 9	*N* = 5
Divorced	*N* = 4	*N* = 1
Widowed	*N* = 6	*N* = 4
Single	N/A	*N* = 2
Unknown	*N* = 1	N/A
Education Completed	M = grade 7	M = grade 7
	(range = no education-grade 10)	(range = grade 5-grade 11)
Number of Children	M = 4.4 (range 0–7)	N/A^a^
Number of Years as a Fish Trader	M = 16.6 (range 5–35 years)	N/A^a^
HIV Status		N/A^a^
HIV-	*N* = 14	
HIV+	*N* = 2	
HIV status unknown	*N* = 4	

^a^information not asked during FDG

### Themes

Template analysis resulted in the following five *a priori* major themes (with emergent sub-themes discussed below): 1) migration and the fish trader business; 2) trauma exposure; 3) psychosocial problems/outcomes as a result of stressors/traumatic events; 4) HIV risk behavior; 5) relationship between stressful life events, psychosocial problems and HIV risk behavior. Summarized data from the FGD and individual interviews can be accessed in the Additional file 1.

### Migration and fish trading business

#### Sub-theme: fish trading as “suffering” and conditions of the river

The majority of participants described the fish trading business and their quality of life as “suffering.” For example, in describing the fish trading business, one participant reported that “it is lack of essential needs like food, clothing, shelter, basic needs, education, and other social amenities needed for survival” (Participant C05). Participants indicated that they would prefer a different occupation, but had no other options for employment. Female fish traders also reported that the occupation requires little more than an introduction to the field, usually from family or friends, and nominal start-up costs, making it a viable option for women with minimal education. One participant acknowledged that she felt little hope in the fish trade business, but had no other options: “Fish is difficult to find and business has gone down. So the worry is how to fend for our families. I only know fish business” (Participant C13).

Participants from the interviews and focus groups felt an immense amount of pressure to provide for their families. Many participants reported being the primary provider for their family, with 31% (*n* = 6) being widows. As such, the effects of bad business and scarcity of fish had negative consequences on the fish trader and her family. Participants reported that when they were not doing well in their business, they experienced more loneliness and ruminated on negative thoughts. One participant indicated:

I do feel lonely. I think about my children and their future. Sometimes I ask myself where I will go with the same business. I do not see progress. I am at the same position ever since I started in 2010. If I can have other things, I could stop and start another business. Sometimes I run at a loss. (Participant C14)

The majority of respondents reported poor living and working conditions at the river in Kafue. One participant described these conditions: “The type of life at the river is a living hell. There is no proper sanitation, and we live like animals” (Participant C09). Interviews revealed that people at the river use contaminated water, which propagates the spread of disease and can be a source of infection. One participant explained that the river is the source of many dangers: “A lot of people die in water, even my child died in water. When people die in water, some bodies decompose before retrieving it. Due to lack of protective clothes, people touch those dead bodies which are a threat to disease” (Participant C07). Participants also feared HIV, sexually transmitted infections, tuberculosis, and malaria, reported as common at the river. Female fish traders discussed the risk of pregnancy, potentially life threatening in these conditions. Lack of nearby services made obtaining medical care difficult. One participant explained that she feels “pity for people who live around the river. The life is miserable. They have no schools, clinics, police posts, and other recreational facilities. Pregnant women deliver children in homes, which are a risk” (Participant C12).

#### Sub-theme: migration patterns and transportation

Participants reported spending between 5 days and 3 weeks away from home at a given time (to obtain fresh fish). The number of times they visited the river varied from two to four times each month. The variability in duration and number of visits was largely unpredictable and beyond the control of the female fish traders. Scarcity of fish, weather, and willingness to have sex with drivers or fishermen were said to influence frequency of visits, transportation time and time spent at the river.

Interview participants with children (*n* = 19) reported that frequent migration adversely affected the well-being of their children. One female fish trader discussed the lack of support for children at the river: “I have worries about the welfare of my child since we don’t have schools at Kafue River. Our children don’t go to school. I think about their future. I have no one in town to support them” (Participant C04). Some participants had the ability to leave children with husbands, older children, or other family members. However, participants without childcare reported that without supervision, children would frequently run out of money for food or school fees.

Frequent and necessary mobility to and from the river was combined with dangerous and unsafe transportation for the majority of participants. One participant reported that “the problem is the way some drivers drive their vehicles. It is a risk to us. But on the river we use speedboats. Even though there is less risk with speedboat, we can’t rule out accidents…” (Participant C11). Longer travel times equated to an increased risk for injury, illness, or death for the fish traders. On the roads, participants reported forced reliance on bus and truck drivers for transport who are under the influence of alcohol or engage in reckless or aggressive driving, often on unpaved and unlit roads. Female fish traders described sitting unrestrained and exposed atop baskets of fish in open-bed trucks. On the water, participants described frequent exposure to animals (i.e. hippopotamuses and crocodiles) and floods. One focus group participant described common experiences in boats: “When boats capsize, all the crewmembers are at risk of perishing. Those who don’t know how to swim, they die. At times, even when one knows how to swim, she is caught by crocodiles” (Focus Group Participant).

#### Sub-theme: competition in the fish trading business

Many participants described negative feelings between fish traders from Lusaka and those from Kafue resulting in competition in the fish trading business. Female fish traders who were willing to have sex with fishermen were assured of receiving fish. One participant gave a synopsis of these relationships: “Sometimes fishermen would opt to give fish to a newcomer just because they want to have sex with her, and when the relationship has developed, it means the fish has to be sold to her first, then the extra is now sold to us” (Participant C13). Thus, if a female fish trader engaged in transactional sex for fish, she would deprive others of being successful in their business.

Female fish traders from the river who were married to fishermen, often blamed fish traders from Lusaka for their marital conflict. Participants from Kafue River (*n* = 9, 45%) expressed anger towards female fish traders from Lusaka who they believed to engage in sexual relations with their husband. One married participant from Kafue reported: “I and others who live in Kafue near the river have husbands. The problem is with the women who come from here in town [Lusaka]. They go out with our men. This situation causes fighting and confusion in our homes,” (Participant C03). Conversely, female fish traders from Lusaka explained that they were helpless in this situation. One participant highlighted:

When you are approached by [fishermen] to have sex with you in exchange for money, then you refuse, they look for ways of punishing you. They organize their friends to swindle the money from you so that you end up stuck in business. The fishermen who got the money would even start trading their fish in a different location. (Participant C09)

Though some female fish traders expressed vulnerability in this situation, there remained a sense of resentment towards women who resort to transactional sex for fish or transportation. Participants reported that women engage in transactional sex because “they lack sense and respect” (Participant C08) and “have no self-control” (Participant C02). Further, there was a belief that female fish traders who engaged in sex for fish did so because they enjoyed breaking up marriages or that they wanted free fish even though they could afford to purchase it.

The competition for fish and resentment among fish traders inhibited the ability to form and maintain friendships and a social network. As reflected by one participant: “I have few friends. Most of the women that do fish business are not honest. They come to get married to fishermen in order to get favors from them” (Participant C06). Another female fish trader discussed relationships with other female fish traders were difficult to maintain because friendships are based on income: “If your business has gone down and stranded, friends avoid you” (Participant C07).

#### Sub-theme: the annual fish Ban and rules of the business

Due to the depletion of fish stocks, the Government of Zambia, under the Fisheries Act of 1994, implements a fish ban ever year for 3 months (December 1^st^ – March 1^st^) across all fisheries in Zambia. The rationale of the ban is to ensure sufficient time for the replenishing of depleted natural resources of the lakes and rivers (i.e. fish). During the fish ban season, it is an offense to engage in any fishing activities or be in possession of fish. As there is generally little economic payoff for female fish traders, the annual fish ban has disproportionately negative consequences on them. Not only does the fish ban impact the fish traders financially, participants reported that they are at risk of imprisonment even if they are in possession of dry fish, which was caught before the fish ban. For example, one participant noted that “[During] fish bans, they always grab items and fish and put us in cells” (Participant C18). Further another participant shared:

During fish ban, we suffer so much because of hunger. The fisheries always confiscate our boats and nets when [we are] caught fishing. We need to be given [a] chance to fish and to be able to buy food for our families, because these are hard times for us at the river. We depend on fish for survival. (Participant C16)

In addition to the fish ban, many fish traders in our study also highlighted structural policies that were put in place at the river which perpetuated lack of power and the subjugated roles of women within the fishing community: “There are rules [related to transactional sex] which chairmen at the river impose on all traders. Such rules contribute to prostitution in fish trade. Women are compelled to associate with fishermen for them to have access to the fish” (Participant C12). In this regard, both the fish ban and imposed rules within the fishing community serve to not only prevent female fish traders from economic success, but place them at an increased risk for HIV infection through necessary transactional sex with fishermen.

### Fish trader-related traumatic events

#### Sub-theme: interpersonal trauma

Female fish traders face risk of gender-based violence such as intimate partner violence and sexual assault at the river and in the home, driving HIV risk exposure and risk of traumatic symptomology. Every participant in the cohort of semi-structured interviews identified at least one case in which they witnessed or heard of a woman being raped at the river. Two women also reported that they themselves had experienced rape. Although not labeled as sexual violence or rape, many participants identified with the term “forced sex” in the context of their marriage. As such, one participant reported her experience: “We women are prone to that [i.e., forced sex] by mostly our husbands” (Participant C07). Many explained that their husbands forced them to have sex when they were “tired.” In line with the perception that the risk of forced sex was common among female fish traders, but sexual violence was not, one participant in the focus group reported:

Sexual violence doesn’t happen in most cases. Fishermen just propose love to us. It’s up to an individual to accept or turn down the proposal. Some women accept sexual relations with fishermen in order to be receiving fish and buying cheap fish from them. Therefore, forcing someone to have sex is not common in fishing camps. In most cases because of overstaying at the river, we do temper with our capital. In so doing we end up getting fish on credit from fishermen in order to balance up the capital. If business did not move well and you fail to pay in good time, that becomes a trap because fishermen demand for sex to compensate for their money. (Focus group participant)

Participants also noted that child sexual abuse was common among female fish traders, with sexual assault and forced marriage being typical forms. One participant recalled her personal experience from childhood: “I was at school and was being kept by my sister, and the man who forced me to sleep with him was being kept [there]. Just that [one time we had sex], I was pregnant. That’s how I got married” (Participant C16). Other fish traders noted that child brides were common at the river: “That trend happens where we go to buy fish at the river. You will find a 12-years-old forced in marriage” (Participant C12).

Study participants also frequently noted instances of interpersonal trauma at the river through theft and assault. Robberies were common at the river and in the market as one participant shared: “From town we load our fridges with ice blocks. Then from the river we load fish in our fridges and load it in trucks. Sometimes thieves follow us like the way we were robbed” (Participant C15). Interpersonal trauma was also reported as a result of conflict from extramarital affairs. As participants mentioned, these relationships had an impact on the entire fishing camp and one participant described her own feelings about the conflict: “Extramarital sex with other people’s husbands… brings fighting and confusions where we buy fish. Those things make me to be afraid…” (Participant C01). One female fish trader explained that her husband’s ex-girlfriend would constantly approach and insult the participant (Participant C05). Further, the participant witnessed the same woman attack her husband with a knife (Participant C05).

#### Sub-theme: non-interpersonal trauma

As previously discussed, female fish traders reported constant worry related to potential injury or death due to travel conditions. In addition to this personal risk, they also described frequently witnessing accidents or death in boats or in trucks. Female fish traders also reported common exposure to floods, which regularly forced them out of their homes. Study participants commonly noted a lack of security associated with frequent migration and the constant fear of not being able to return home. For example, it was indicated that “at the river we live a nomadic type of life. During the floods, we migrate to higher land…Now we have heard that our house have been demolished so we don’t know how we shall survive” (Participant C16).

Similarly, loss of shelter was also attributed to frequent exposure to fires at the river as temporary shelters are mostly made of grass. About half of participants experienced a fire incident and nearly every participant had either witnessed or heard of fire affecting someone at the river. One female fish trader explained that “most people who live in grass houses are prone to [fire]. Some fires are as a result of sabotage by people who have been offended” (Participant C05). Participants reported that men and women often used temporary shelters to engage in transactional sex, thus placing them at risk of arson from another fish trader who was competing for fish with the fisherman.

Approximately three-quarters of participants in the interviews reported that a close friend or family member died. The death of loved ones, especially a husband and/or parent(s), not only took an emotional toll on the female fish traders, but also had adverse financial implications. Without a dual income or the support from parents, female fish traders struggled to survive. For example, a female fish trader described how the death of her husband affected the long-term wellbeing of their whole family: “I think about the education of my children and their survival, whether I will manage to take care of them since I am now a widow” (Participant C20).

### Psychosocial outcomes

#### Sub-theme: outcomes consistent with the western conceptualization of PTSD and depression

Symptoms consistent with post-traumatic stress disorder were commonly indicated by study participants. Arousal symptoms of PTSD were described by a number of female fish traders who frequently experienced a quick temper and irritability. For example, one participant noted uncontrollable angry outbursts that felt unrelated to any particular event: “Sometimes, even when [I] am not reminded, I do feel that pain and hate. I don’t know how that comes to me. I do have tempers” (Participant C08). Sleep disturbances were also common among female fish traders with *n* = 9 participants (45%) reporting an inability to sleep accompanied with worry and rumination of stressful situations.

PTSD symptoms of intrusion were also reported, especially distressing trauma-related dreams and nightmares. The majority of participants from the interviews described ongoing nightmares related to traumatic events (*n* = 14, 70%). Participants described vivid nightmares regarding dead relatives, being chased or threatened, and sexual assault. For example, one participant reported nightmares related to death: “I do dream about dead people coming to me” (Participant C10). Still another shared a nightmare related to sexual assault and violence: “Just after prayers, I dreamt of a dog trying to sleep with me” (Participant C15).

Symptoms of avoidance, especially avoiding thoughts and memories related to traumatic events were common among the participants. Participants commonly described purposefully staying busy in order to avoid thoughts of stressors or traumatic events. One participant reported that her strategy is to “normally get busy with other people to avoid thinking about bad things which happened to me” (Participant C08). Avoidance of external reminders was more difficult for participants as they were frequently exposed to the location of traumatic events at the river. A participant stated that “we always witness friends that die while we had been with them a few hours or minutes ago…but there is nothing we can do but to continue with business in order to sustain our families” (Participant C19).

PTSD symptoms related to cognitive and affective alterations were not uncommon, as participants reported feelings of negative expectations through a loss of hope, self-blame and persistent feelings of guilt. For instance, one female fish trader took responsibility for her husband’s extramarital affair: “In most times [I] blame myself, especially in my marriage. I feel maybe I am at fault. That’s why my husband moves away from home to look for women. The guilt comes sometimes” (Participant C05).

Overlapping with the loss of hope reported by many participants was suicidal ideation, other symptoms resembling depression (e.g. decrease in appetite) and symptoms of grief. One participant reported that “there was a time when my husband died. My mother also died. After that, my father also died. It was death after death within a short space of time. I was hoping to die. I even stopped eating food. I felt there was no future for me” (Participant C19). A majority of participants from the interviews reported experiencing the death of a loved one (*n* = 17, 85%) frequently as a result of an AIDS-related illness. As a means to cope with ongoing stressors and trauma, one participant noted “some people plan to kill themselves when they face problems and challenges. Others would even contemplate to kill all [his/her] children and the spouse before taking their lives” (Focus group participant).

#### Sub-theme: substance abuse

Use of substances to cope with stressors was a frequently reported. Participants reported a range of substances including alcohol and drugs. While substance use behavior was frequently reported by participants, the majority of participants denied use themselves. Among those participants who endorsed drinking alcohol, did so as a past behavior. One participant explained her regret for having ever engaged in substance use: “I do feel bad when I remember the times I used to drink. I feel that I wasted time instead of serving God,” (Participant C15). Several participants also reported use of other substances at the river, such as heroin, cocaine, marijuana and inhaling local synthetic drugs.

#### Sub-theme: local idioms of distress

As described earlier, study participants endorsed and described a range of symptoms associated with western conceptualizations of mental health symptoms and diagnoses, specifically PTSD and depression. However, many participants indicated psychosocial outcomes as a result of trauma and ongoing stressors that do not encompass a western term of mental health. For example, many participants reported problems related to the heart, thinking too much, and experiencing a lack of peace. In describing feeling assaulted by another female fish trader, one participant shared that “she always comes to insult me. I always fall sick every time. Now I don’t know whether it’s the torture or maybe I think too much. I don’t normally have peace, always thinking on how to get rid of her in my mind” (Participant C05). Another participant reported experiencing symptoms related to the heart as well as symptoms similar to anxiety: “My child was very sick and my business went down. Things were just hard for me. I was tortured in my mind and I developed anger and hate in my heart. That resulted into sweating and fast pumping of the heart” (Participant C06). In examining the nature of psychosocial problems related to the heart a focus group member described what it means to have *no peace*: “Someone who has no peace has problems and is anxious and in a panicked mood. The mind and heart is unstable,” (Focus group).

### HIV sexual risk behavior

#### Sub-theme: transactional sex

Similar to use of substances, during the semi-structured interviews, only one female fish trader reported a history of transactional sex with a fisherman for fish while nineteen participants denied engaging in transactional sex. Yet, all participants stated that transactional sex was commonplace and necessary in the fish trading business. For example, one participant noted:

Some women just come with transport money to the river and start selling their bodies in order to buy fish… It’s common among women fish traders [to have both fishermen and drivers as their sexual partners]. They do that in order to be having free fish and free transport. (Focus group participant)

Though most participants did not endorse transactional sex themselves, many participants reported that they had an intimate relationship with a married fisherman who also had other sexual partners at the river, a major driver of HIV infection. Many also reported concern about risk of HIV infection from their spouse. One participant described the potential risk of HIV from her husband related to transactional sex at the river: “These [women who sell their bodies for fish and money] do not select who to sleep with, so I am worried about my husband’s loose life to women” (Participant C05). Another participant explained the effects of promiscuity at the river in that “one man can have sex with a lot of women so diseases are all over. They just exchange women. There is no permanent marriage” (Participant C09). Lack of disclosure of a positive status from their partner was also reported among participants, increasing HIV risk. One participant shared that her “[ex-husband] was a drunkard and was HIV positive. My husband had to hide his status for 1 year without disclosing to me and was refusing to use condoms” (Participant C15).

#### Sub-theme: condoms and HIV testing

Although participants were aware men have multiple and concurrent sex partners, women reported inconsistently using condoms. Participants commonly reported that men would be more likely to use condoms with their girlfriends rather than their wives. All of the married participants reported lack of condom use with their husband as it was a sign of infidelity or lack of trust between partners. Participants also frequently reported that condoms were rarely used as it diminished sexual pleasure. For example, one participant remarked that having sex with a condom is “like eating a sweet with its packaging material” (Participant C10). Stigma was also associated with condom use with one focus group participant reporting that many fish traders “avoid using condoms for fear of cited to be a prostitute” (Focus group participant).

HIV testing was also uncommon among the female fish traders in the current study with 20% (*n* = 5) of the interview sample reporting an unknown HIV status. Respondents reported that people at the river are likely to take an HIV test only when they were already sick. One participant reported that people do not get tested because they “fear to be declared positive. Some people feel embarrassed to start going to collect ARVs [antiretroviral] at the clinic” (Participant C19). Participants reported stigma and shame related to people living with HIV (PLWH) and a general sense that a positive status was a result of careless actions. Blame towards PLWH for spreading HIV among the fishing community was also a common sentiment.

### Stress/trauma, psychosocial outcomes and HIV risk behavior

Among the interviews and focus group discussion, there was evidence in support of the conceptual model indicating a relationship between migrant-related stress, trauma, psychosocial problems and HIV sexual risk behavior. For example, a participant in the focus group explained the relationship between stressors, isolation and HIV sexual risk: “Women get involved in extramarital affairs because family members don’t give them support. Some are orphans, others are widows and divorcees. These people are vulnerable and have nowhere to go to get help and support” (Focus group participant). Some participants described excessive drinking and sexual risk behaviors as self-destructive behaviors, which enabled them to avoid problems: “They would just sell fish and go back [to the river] without taking care of the welfare of the family. That worries me because they have little children, these women engage in sexual activities and beer drinking” (Focus group participant). Similarly, during the focus group, it was noted that “women have demons that compel them to drink beer and to have more sexual partners…to avoid worries it’s better to have more sexual partners. But others drink to avoid loneliness” (Focus group participant). This quote highlights HIV sexual risk behavior as a coping mechanism to avoid trauma symptoms, as well as excessive alcohol use as a means to numb trauma symptoms, with subsequent HIV sexual risk.

Consistent with the results related to transactional sex and substance use, none of the participants reported support for the relationship between trauma and HIV sexual risk personally. However, many participants acknowledged the nature of the relationship as commonplace among female fish traders in general. For example, one woman shared why she believes that people engage in HIV risk behaviors: “To some people it’s their heart, to some its lust, and others it’s because of the suffering which they face in their homes.” (Participant C12). Conversely, many participants reported that they experienced ongoing trauma and stress at the river without subsequent HIV sexual risk behavior. One participant reported “if [I] live carelessly, I may end up contracting HIV and STIs which is sad and retrogressing as a breadwinner,” (Participant C20). Other participants noted that they suffered many hardships at the river but their religious beliefs helped them cope with their problems and trauma symptoms. One participant noted “I don’t have bad dreams. I pray” (Participant C07).

## Discussion

### Theory of gender and power on women’s health

This is the first known study to examine the experience of trauma, psychosocial problems, and HIV sexual risk among the key-affected female fish trading population in the Kafue Flatlands of Zambia. Our findings are consistent with the emergent literature related to the highly gendered political and social structure of fish trading in fishing communities of sub-Saharan Africa [4, 16]. Further, our findings provide support for the overarching framework guiding this study, the theory of power and gender on women’s health [22], in that the sexual division of labor, sexual division of power and the structure of cathexis related to gender norms and roles are upheld within the fishing community of Kafue, and demonstrates how risk of HIV, substance use, and trauma symptomology is generated. In the sexual division of labor, women are relegated to the position of fish trader, a difficult occupation with very little payoff. This is a reflection of a complex masculine social system, which subordinates women and constrains their life chances. In Zambia, where historically, women are ranked lowly with limited access to education compared to men [36], it is plausible that women would lack sellable skills and as such, would have problems finding alternative sources of livelihood. Moreover, with over 30% of the study sample reporting widowhood, these limited economic opportunities and simultaneous pressure to provide financially for their families further exacerbates stressors and challenges for women, shaping their HIV risk behaviors.

In the sexual division of power, female fish traders in our study all indicated the necessity to engage in transactional sex with fishermen and truck drivers for economic livelihood. The division of power was also indicated by the high frequency of forced sexual relations with their husbands. On the structural level, the enforced “rules of business” perpetuate transactional sex to gain access to fish for the traders, which not only increases HIV risk, but competition among fish traders, creating an environment of isolation and lack of social support. In addition, lack of access to services, prevalent concurrent sex partnerships among men, and an inability to negotiate condom use with high-risk main and casual partners contributes to HIV risk among fish traders.

### Trauma, violence and ongoing daily stressors among female fish traders

According to the structure of cathexis, disparities resulting from strict gender norms and beliefs among female fish traders are manifested in the psychosocial domain as personal risk factors for HIV risk [22]. Placed within the domain of social exposures and personal risk factors is the conceptual model further explaining the pathway of trauma and violence to HIV sexual risk behavior [22, 24]. Female fish traders in our sample indicated a number of social exposures and personal risk factors related to the dangerous, demeaning and difficult job of fish trading. Not only were our findings consistent with previous literature that has indicated high rates of sexual violence within fishing communities in Zambia [18], but highlighted the frequent exposure to both interpersonal *and* non-interpersonal trauma and violence within the fishing communities. Exposure to and witnessing of intimate partner violence, forced sex, assault and robberies at the river, deadly boating and road accidents, fires, floods, imprisonment, and AIDS-related deaths were overwhelmingly common among our study sample. Further, female fish traders reported ongoing daily stressors related to the fish trading business, e.g., lack of childcare and health services due to mobility and remote locations of the river, financial stressors due to the scarcity of fish, the fish ban and competition among fish traders.

### Psychosocial problems: western conceptualizations and local trauma symptoms

Fish traders in our study reported severe and ongoing psychosocial problems related to trauma exposure and daily stressors. Although we only probed for specific symptoms of PTSD, mental health problems were consistent with PTSD (e.g., avoidance, intrusion, nightmares, self-blame), as well as depression (e.g., hopelessness, lack of appetite, suicidal ideation), anxiety (e.g., excessive worry, racing heart), complicated grief (e.g., intense feelings of loss and sadness), and substance abuse (e.g., alcohol abuse, local synthetic drugs, marijuana). These findings are consistent with other studies among trauma-affected populations from sub-Saharan Africa [37, 38]. In addition, we found that local trauma symptoms related to a “lack of peace,” “thinking too much,” and symptoms related to the heart (i.e., “anger in the heart, hatred in the heart, injury to the heart”) were common. Symptoms consistent with western syndromes, as well as local symptoms, both accurately reflecting outcomes of trauma and migrant-related stressors, are in line with previous research conducted in non-western LMIC [38–41]. These findings indicate that western conceptualizations of mental health problems can be relevant, but the local expression of symptoms must be taken into account to obtain a full picture of suffering within non-Western LMIC. Future studies should examine the extent of the impact of psychosocial problems indicated in our study on overall functioning and productivity of female fish traders.

### HIV sexual risk behavior: the pathway between trauma and HIV risk

Consistent with other studies conducted within fishing communities in sub-Saharan Africa [4, 16, 18], our study found that HIV sexual risk behavior was common among female fish traders. Interestingly, similar to a study conducted in a fishing community in Malawi [4], female fish traders in our study individually reported inconsistent and/or lack of condom use with their main partner and high risk partners, but only reported transactional sex with fishermen and truck drivers as a common sexual risk behavior of *other* female fish traders. Our study was also in line with previous research that has indicated a relationship between substance use and HIV sexual risk behavior in female fish traders in sub-Saharan Africa [16, 42], although in our sample participants only disclosed substance use as a behavior engaged in by others. The lack of disclosure of substance use and transactional sex among female fish traders in our study may have been accurate, or possibly influenced by the stigma associated with these behaviors in Zambia and potential harsh penalties.

**Fig. 1:**
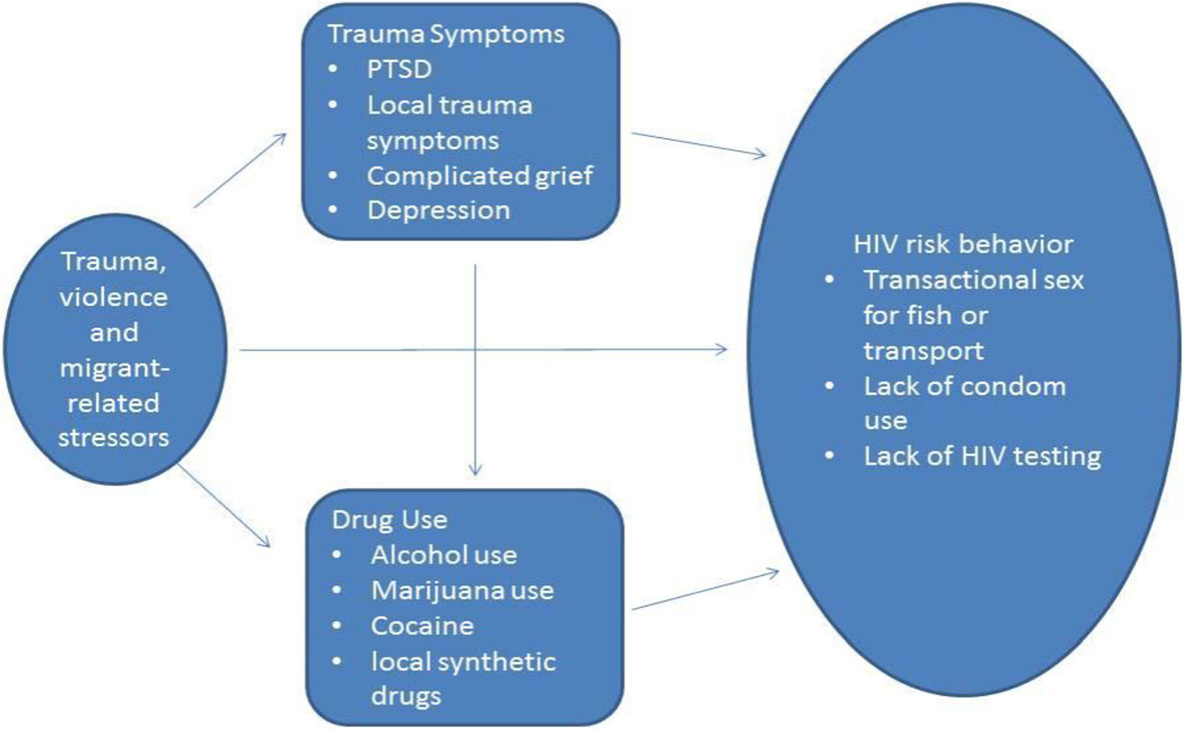
*Conceptual Framework of Co-occurring Issues Associated with HIV Risk Among Female Fish Traders* adapted, Miller, [24]

Participants from our study recognized the relationship between trauma and HIV sexual risk behavior among female fish traders. Participants reported that trauma contributes to an increase in mental health symptoms and maladaptive coping mechanisms, such as substance use, which subsequently leads to HIV risk (see *Fig.* 1 *below: Conceptual Framework of Co-occurring Issues Associated with HIV Risk Among Female Fish Traders* adapted, Miller, [24]). On the other hand, some participants reported that trauma and violence did not lead to HIV sexual risk. Protective factors, such as resilience and healthy coping mechanisms, should be explored in future research as potential mediators between trauma and HIV risk behavior among this population [43]. In our study, the use of religion, prayer, and “offering hardships up to God” were commonly reported as a means to cope with the difficult life associated with the fish trading business. In addition, female fish traders in our study frequently referred to transactional sex, substance use and multiple partners as the work of the “devil.” Perhaps reliance on religion as a coping mechanism serves as a protective factor against the development of mental health symptoms, substance use, and subsequent HIV sexual risk behavior. Future quantitative studies should examine the relationship between trauma and HIV risk distinguishing type of trauma and the role of religion as a coping mechanism in order to disentangle the nature of the relationship among female fish traders in Zambia*.*

Type of trauma experienced could also help explain the inconsistent findings. Research in western contexts has indicated that betrayal trauma, or interpersonal trauma where the individual must rely on the perpetrator for financial or emotional support (e.g. sexual violence from a fishermen with whom the fish trader relies on for financial support), may result in dissociative symptoms as an adaptive mechanism to preserve the relationship for survival [27, 44]. Dissociative symptoms and substance use as numbing mechanisms, while potentially adaptive within the relationship, have adverse consequences for long term functioning [45] and an individual’s ability to discern risk in the environment, increasing HIV sexual risk behavior [24] and risk of re-victimization [27]. On the other hand, the pathway from non-interpersonal trauma to HIV sexual risk among female fish traders may be less likely to include dissociative symptoms and substance abuse decreasing HIV sexual risk. Future research should examine this difference and attempt to determine perceptions of betrayal trauma vs. non-interpersonal trauma among female fish traders in Zambia and the potential pathway to mental health, substance use and HIV risk outcomes from each.

### Strengths and limitations

There are a number of strengths and limitations of the current study which should be noted. This is the first study to examine the relationship between trauma, mental health, substance use, and HIV risk behavior among the key-affected and vulnerable population of female fish traders of Zambia. Our study used qualitative methods which enabled us to obtain rich descriptive data related to the experiences of female fish traders. We also had multiple reviewers of the data, including a native Zambian to ensure cultural relevance of our findings. In addition, we used different methods of data collection (i.e., semi-structured interviews and a focus group discussion), which allowed for confirmation and further probing for accurate results. Along those lines, we used open-ended questions related to trauma symptoms and specific questions related to PTSD symptoms allowing for the assessment of both established western mental health and psychosocial problems and local symptoms of distress. In our analysis, we ensured that all participants’ voices were represented in the results with each interview participant mentioned at least once reducing bias in the findings.

At the same time, there are a number of limitations that should be mentioned. All of the participants except one discussed transactional sex and substance use in relation to others, but did not indicate this behavior personally. Although this is in line with other research among female fish traders in sub-Saharan Africa [4], future studies should examine ways of community engagement and use of alternative methods of data collection to potentially reduce self-reporting bias. In our study, a male interviewer conducted all of the interviews which could have compromised the comfort levels in disclosing sensitive and stigmatizing behaviors related to sexual behaviors and substance use among female fish traders. Although a female interviewer may have obtained different results, the research assistant we chose to work with was most qualified for the position. Further, although we took steps to ensure methodological rigor to obtain culturally relevant results, cultural differences could potentially have influenced our findings. Specifically, there are no equivalent words in Nyanja for many of the mental health symptoms, which we explored in our study (e.g., intrusion, avoidance, exaggerated startle response). As such, translation at times required a detailed description in local terms of the mental health construct, potentially biasing the results due to the participant endorsing a description of a symptom which was not the exact equivalent of the mental health term. Finally, as our study employed qualitative methods with purposive sampling methods, caution should be taken in the transferability of the findings to other contexts. Though we attempted to recruit women from a wide age range, this sampling method resulted in a sample of older women. The literature notes that risk for HIV infection and HIV risk behavior is higher in Zambia among young women compared to older women [10]. The findings in this paper represent HIV risk in older women and while these findings cannot be ignored, it should be noted that these results do not represent younger women whose risk for HIV infection may be higher. In addition, in the current study we were interested in examining the relationship between trauma and HIV sexual risk behavior but acknowledge the limitation that we did not specifically assess the status of sexually transmitted infections. Future quantitative methods should examine this among female fish traders in Zambia.

## Conclusions

Findings from our study reveal the need to develop individual, community, and policy level interventions in order to adequately reduce HIV risk behavior among female fish traders in Zambia. On the individual level, our study demonstrates the critical need to develop culturally-relevant, gender-based and trauma-informed HIV prevention interventions that address issues of mental health, substance use, and HIV sexual risk behaviors. A recent review in the U.S. of the syndemic occurrence of HIV and trauma lends further support for increased attention and integrated care to address these co-occurring issues, emphasizing trauma-informed patient care and additional training and support of providers [46]. In addition, HIV prevention efforts should be developed for female fish traders who are living with HIV and at risk for HIV in order to reduce HIV transmission risk behavior and to keep fish traders who are negative healthy. Access to drug treatment and education about the impact of drug and alcohol use on health is critically needed among female fish traders. On the community level, the development of innovative HIV health services that include the potential use of mobile services and/or use of mobile technology should be tested at the river to address the needs of this highly mobile population. Further, as many female fish traders from our study have children, feasible alternatives to regular schools at the rivers and lakes should be considered to increase access to education. On the macro level, findings from our study indicate the need for governments to provide alternative means of livelihood during the fish ban, such as farming or educational opportunities to increase economic means for women involved in the fish trading business. In addition, government provided safety provisions, such as life vests and adequate housing at the river can potentially reduce boating accidents and deaths by fire/flood in makeshift houses made of grass. Finally, culturally relevant and feasible changes in the gender and power structure of the fishing community are critical and should be considered at the policy level. Looking forward, it is imperative that researchers and interventionists engage male and female fisher folk in the process of reducing trauma, mental health problems and HIV risk among this key-affected population.

## Additional file

Additional file 1: "Focus group quotes". "Interview and FG demographics". "Quotes from themes from analysis". (ZIP 60 kb)

## Abbreviations

FGD: Focus group discussion
HIV: Human Immunodeficiency Virus
LMIC: Low and middle income countries
PLWH: People living with HIV
PTSD: Post Traumatic Stress Disorder
